# Hyperinsulinemia shifted energy supply from glucose to ketone bodies in early nonalcoholic steatohepatitis from high-fat high-sucrose diet induced Bama minipigs

**DOI:** 10.1038/srep13980

**Published:** 2015-09-11

**Authors:** Shu-lin Yang, Ji-han Xia, Yuan-yuan Zhang, Jian-gao Fan, Hua Wang, Jing Yuan, Zhan-zhao Zhao, Qin Pan, Yu-lian Mu, Lei-lei Xin, Yao-xing Chen, Kui Li

**Affiliations:** 1 State Key Laboratory of Animal Nutrition, Institute of Animal Sciences, Chinese Academy of Agricultural Sciences, No.2 Yuanmingyuan West Road, Beijing, 100193, P.R. China; 2 College of Veterinary Medicine, China Agricultural University, No.2 Yuanmingyuan West Road, Beijing, 100193, P.R. China; 3 Department of Gastroenterology, Shanghai Key Laboratory of Children’s Digestion and Nutrition, Xinhua Hospital, Shanghai Jiaotong University School of Medicine, Shanghai, 200092, P.R. China; 4 Department of Oncology, The First Affiliated Hospital of Anhui Medical University, Hefei, 230032, Anhui, P.R. China; 5 College of Animal Science, Yangtze University, Jinzhou, 434023, Hubei, P.R. China

## Abstract

The minipig can serve as a good pharmacological model for human subjects. However, the long-term pathogenesis of high-calorie diet-induced metabolic syndromes, including NASH, has not been well described in minipigs. We examined the development of metabolic syndromes in Bama minipigs that were fed a high-fat, high-sucrose diet (HFHSD) for 23 months, by using histology and serum biochemistry and by profiling the gene expression patterns in the livers of HFHSD pigs compared to controls. The pathology findings revealed microvesicular steatosis, iron overload, arachidonic acid synthesis, lipid peroxidation, reduced antioxidant capacity, increased cellular damage, and inflammation in the liver. RNA-seq analysis revealed that 164 genes were differentially expressed between the livers of the HFHSD and control groups. The pathogenesis of early-stage NASH was characterized by hyperinsulinemia and by *de novo* synthesis of fatty acids and nascent triglycerides, which were deposited as lipid droplets in hepatocytes. Hyperinsulinemia shifted the energy supply from glucose to ketone bodies, and the high ketone body concentration induced the overexpression of cytochrome P450 2E1 (CYP2E1). The iron overload, CYP2E1 and alcohol dehydrogenase 4 overexpression promoted reactive oxygen species (ROS) production, which resulted in arachidonic and linoleic acid peroxidation and, in turn, led to malondialdehyde production and a cellular response to ROS-mediated DNA damage.

Nonalcoholic fatty liver disease (NAFLD) is a growing global health problem^1^ and has a disease spectrum ranging from simple steatosis to nonalcoholic steatohepatitis (NASH), liver fibrosis, cirrhosis, and hepatocellular carcinoma^2^. The pathogenesis of NASH is not completely understood. The “two-hit” hypothesis was proposed as an initial model for the pathogenesis of NASH^3^. The “first hit” is hepatic steatosis due to triglyceride accumulation. The “second hit” comprises several liver injury processes, including oxidative stress, mitochondrial dysfunction, and inflammatory cytokine production. These processes can lead to inflammation and fibrosis. Based on substantial evidence, a modified “two-hit” hypothesis proposed that free fatty acids (FFAs) derived from diet, lipolysis, or *de novo* lipogenesis play a direct role in promoting oxidative stress and inflammation-mediated liver injury, whereas the esterification of FFAs to triglycerides for storage and export can function as a protective mechanism against FAA accumulation^4^,^5^. The “multiple parallel hits” hypothesis considers the central role of gut-derived and adipose tissue-derived factors in liver inflammation. This hypothesis^6^ proposes that endotoxins from the gut trigger the innate immune response and that adipocytokines (adiponectin, leptin) and cytokines (TNFα or IL-6 and others) from adipose tissue play a critical role in NASH, beyond lipotoxicity. The multiple parallel hit hypothesis of NASH is based on the increases in serum FFAs due to insulin resistance (IR) in adipose tissue^7^. Recent studies have highlighted the important roles of iron overload^8^, of alcohol produced by the intestinal bacteria of obese mice and human patients^9^ and of high ketone body concentrations arising from insulin resistance^10^ in the production of the reactive oxygen species (ROS). The molecular interplay between Δ5/Δ6 desaturases and long-chain fatty acids leads to lipid peroxidation and inflammatory injury in hepatocytes^11^. However, there are no studies involving all of the above characteristics in a rodent model or patient, especially for early stage NASH pathogenesis, when hepatic steatosis and serum FFAs are lower and hyperinsulinemia is observed.

NASH can develop and progress over decades. Because of the ethical constraints in obtaining human liver tissue, the quality and interpretation of longitudinal data from humans are inevitably limited. Instead, many animal models are used to study the mechanisms of NASH. Extensive reviews have discussed the advantages and disadvantages of the current diet-induced or genetic models of NAFLD^12^,^13^,^14^ and have concluded that no single animal model can mimic the full spectrum of human NAFLD. Thus, the development of animal models that can accurately mimic the metabolic changes that are characteristic of NASH is crucial for gaining a better understanding of its mechanisms and for designing novel diagnostic and therapeutic strategies for its treatment^15^. Rodent models have been widely used in NASH research because the rodent genome has been sequenced, and genetic modification in these models is easily accomplished. However, differences in lipid metabolism properties between rodents and humans have prevented the translation of rodent data into clinical practice^16^. The pig is an exceptional model organism for obesity and metabolic syndrome research because its metabolic features, cardiovascular systems, and omnivorous habits are similar to those of humans^17^. Furthermore, advances in porcine genomics, proteomics, and genetic modification techniques will facilitate the use of pig models in extensive applications in the biomedical field^18^. The swine breeds Gottingen, Yucatan, and Ossabaw have been used in the research of obesity-related diseases^19^,^20^,^21^ and display central obesity, insulin resistance, and dyslipidemia within 12 months, depending on their specified diets. However, only the Ossabaw minipig, which has undergone natural selection for a thrifty genotype^22^, has shown the characteristics that are typical of steatohepatitis when fed modified atherogenic diets with high fructose and modestly reduced choline concentrations. In addition, a study by Chen *et al.*^23^ showed that the Chinese Bama minipig showed metabolic syndromes, including obesity, insulin resistance, and increased levels of fasting serum glucose, insulin, serum triglycerides (TG), total cholesterol (TC), high-density lipoprotein cholesterol (HDL-C), and low-density lipoprotein cholesterol (LDL-C) when fed a high-fat, high-sucrose diet (HFHSD) for eight months.

Although NASH has been observed in Ossabaw miniature swine fed a diet with lower choline content, it remains unknown whether a long-term high-fat, high-calorie diet can induce NASH in miniature pigs. The Bama minipig has been a closed colony for more than 20 years in China and is widely used in medical research. In this study, Bama minipigs were fed a HFHSD for 23 months and monitored for the development of metabolic syndrome and NASH using serum biochemistry, hepatic profiles, histopathological approaches, and gene expression profiling of the liver from HFHSD pigs. Comprehensive analyses of these data revealed that the pathogenic characteristics of early stage NASH include the following: *de novo* synthesis of fatty acids and nascent TG deposition, energy supply shift, reactive oxygen species (ROS) production, lipid peroxidation, cellular damage and inflammation.

## Results

### Body weight

**Figure 1:**
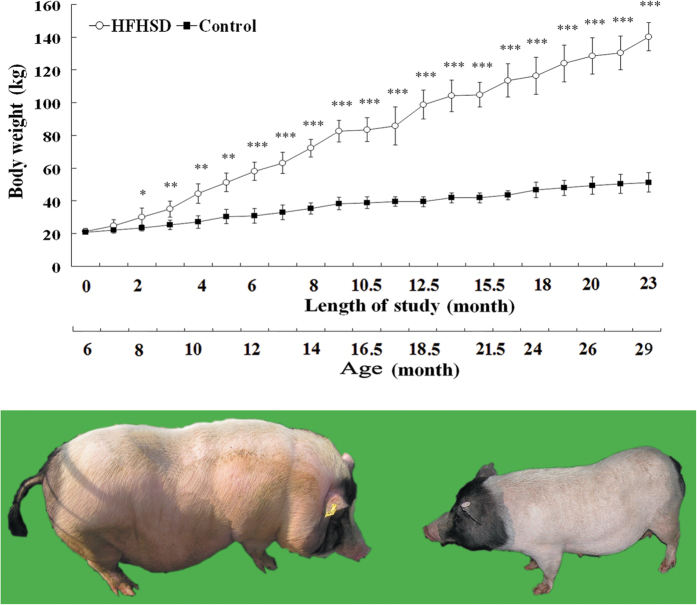
Average body weights of the HFHSD and control groups during the 23-month feeding period. *P < 0.05, **P < 0.01, ***P < 0.001 indicate the level of significance of differences between the two groups (top graph). The bottom graph shows that the HFHSD-fed Bama minipigs (left) are significantly obese relative to the control pigs (right). The image and the figure were produced by Shu-lin Yang.

After 23 months on the prescribed diets, the average body weight of the HFHSD and control groups increased from 20.83 ± 1.27 kg and 21.38 ± 1.74 kg to 140.28 ± 8.52 kg (P < 0.01) and 51.30 ± 5.85 kg, respectively (Fig. 1 and Table 1).

### Triglyceride and cholesterol levels

**Figure 2:**
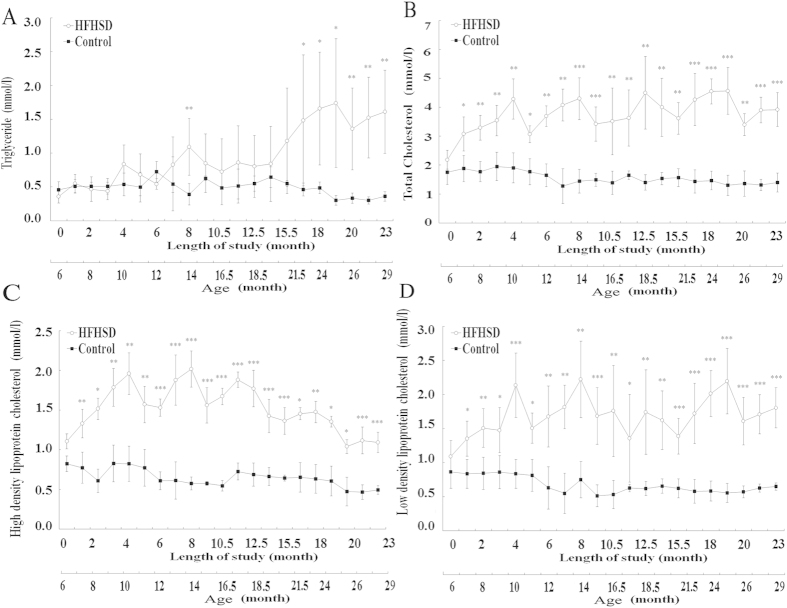
Comparison of serum triglyceride and cholesterol levels in the HFHSD and control groups during the study period. Triglyceride (**A**), total cholesterol (**B**), high-density lipoprotein cholesterol (**C**) and low-density lipoprotein cholesterol (**D**). *P < 0.05, **P < 0.01, ***P < 0.001 indicate the level of the significance of differences between groups.

For the control group, no significant changes in TG, TC, HDL-C, or LDL-C were observed throughout the entire rearing period. For the HFHSD group, the TG levels began increasing at 12 months and were significantly higher at 16.5 months than those of the control group (Fig. 2A). TC, HDL-C, and LDL-C increased rapidly during the initial 4 months. Subsequently, HDL-C fluctuated between months 4 and 12 and eventually decreased after month 12 (Fig. 2C). LDL (Fig. 2D) and TC (Fig. 2B) fluctuated from the fourth month to the end of the study period. TG, TC, HDL-C, and LDL-C were significantly higher in the HFHSD group than in the control group, when the pigs were sacrificed (Table 1).

### Insulin resistance and associated islet enlargement and hyperinsulinemia

**Figure 3:**
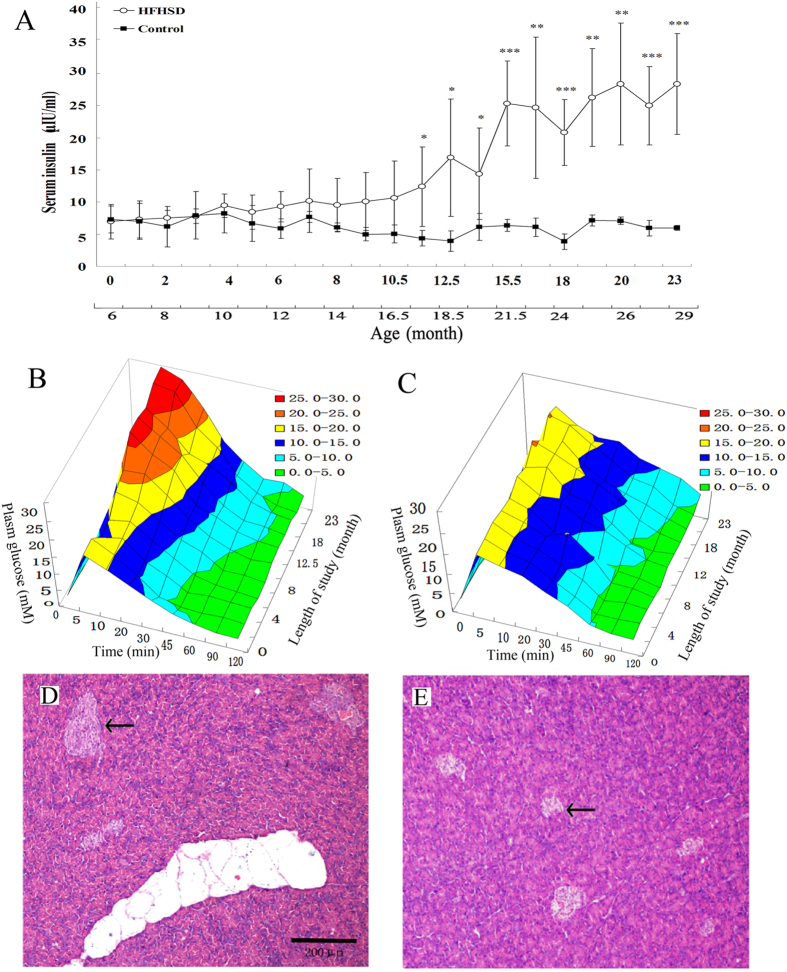
Insulin resistance and associated islet enlargement and hyperinsulinemia. Fasting insulin of the HFHSD and control groups detected in the study period (**A**). The intravenous glucose tolerance tests for the control (**C**) and the HFHSD (**B**) groups. Histological and morphometric analyses of hematoxylin and eosin (**H**,**E**)-stained sections show that the pancreatic islets of the HFHSD group (**E**) are enlarged compared to those of the control group (**D**). *P < 0.05, **P < 0.01, ***P < 0.001.

During the entire rearing period, the fasting glucose did not significantly change in the HFHSD or control groups. The fasting serum insulin remained relatively stable in the control group but began to increase in the HFHSD group at 12 months and reached 28.32 ± 7.84 μIU/L at 23 months (P < 0.01) (Fig. 3A). As shown in the intravenous glucose tolerance test (IVGTT), the peak serum glucose and recovery time following injection began to increase at 12 months in the HFHSD group (Fig. 3B). No significant changes in the peak and recovery times were observed in the control group during the rearing period (Fig. 3C). Histopathology findings revealed that the pancreatic islets were substantially larger in the HFHSD group (Fig. 3D) than in the control group (Fig. 3E). Moreover, the number of pancreatic islets with a cross-sectional area of greater than 10,000 μm^2^ was significantly higher in the HFHSD group than in the control group (Table 1). The Insulin Resistance Index of the HFHSD group was significantly higher than that of the control group during the last seven months of the study (Figure S1), which indicates that the HFHSD group had lower insulin sensitivity than the control group. These results indicate that 1) the enlarged islets resulted in hyperinsulinemia in the HFHSD group, maintained fasting serum glucose within the normal range during the study period, and reduced glucose levels quickly during the IVGTT; and 2) insulin resistance developed in the insulin-targeted tissues or organs.

### Hepatic fatty acid composition, Fe levels, lipid peroxidation, antioxidation, and inflammation

Of the primary fatty acid components in the livers of the sacrificed pigs (Table 1), linoleic acid was the only dietary-derived fatty acid that was significantly lower in the HFHSD group than in the control group. The other dietary-derived fatty acids, except myristoleic acid, were increased in the HFHSD group relative to the control group (P < 0.05, except myristoleic acid). Furthermore, the liver-synthesized arachidonic acid was 2.58 times higher in the HFHSD group than in the control group (P < 0.05). The β-hydroxybutyrate was significantly increased in the HFHSD group (P < 0.01, Table 1) compared with the control group. However, acetoacetate concentrations were not different between the two groups. The serum and hepatic ferrous iron levels of the HFHSD group were 1.54 and 1.87 times higher than those of the control group (P < 0.05), respectively. Because lipid deposition and Fe overload can promote fatty acid peroxidation, malondialdehyde content (the classic marker of lipid peroxidation) was determined and shown to be 3.56 times higher in the HFHSD group than in the control group (P < 0.05, Table 1). This increase was accompanied by significant decreases in serum antioxidant superoxide dismutase, glutathione peroxidase, and total antioxidant capacity in the liver. Moreover, the percentage of inflammatory cells was increased significantly in the HFHSD group (Table 1). These results indicate that arachidonic acid synthesis and Fe overload may produce oxidative stress and inflammatory responses in the HFHSD group.

### Hepatocytic histopathological findings and grading

**Figure 4:**
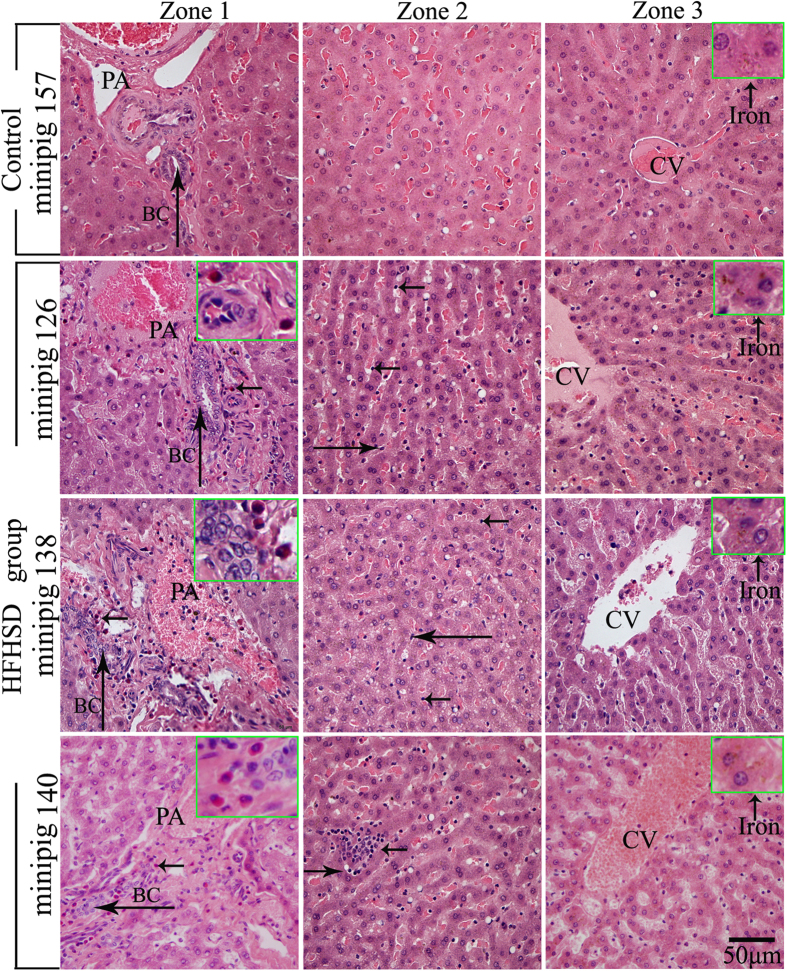
Histopathological changes in the livers of each study group. Control: Histological sections from the control pigs (minipig 157). Histological sections from the HFHSD pigs (minipig 126, minipig 138, minipig 140). Zone 1: connective tissue and bile canaliculus hyperplasia (long arrows) and a large number of eosinophils (short arrows). Zone 2: severe inflammatory cell infiltration in the dilated hepatic sinusoid of the HFHSD livers, primarily Kupffer cells (long arrows) and the lymphocytes (short arrows). Zone 3: mild iron overload in the hepatocytes of HFHSD livers. CV: central venous; PA: portal area; BC: bile canaliculus. Scale bar = 50 μm.

**Figure 5:**
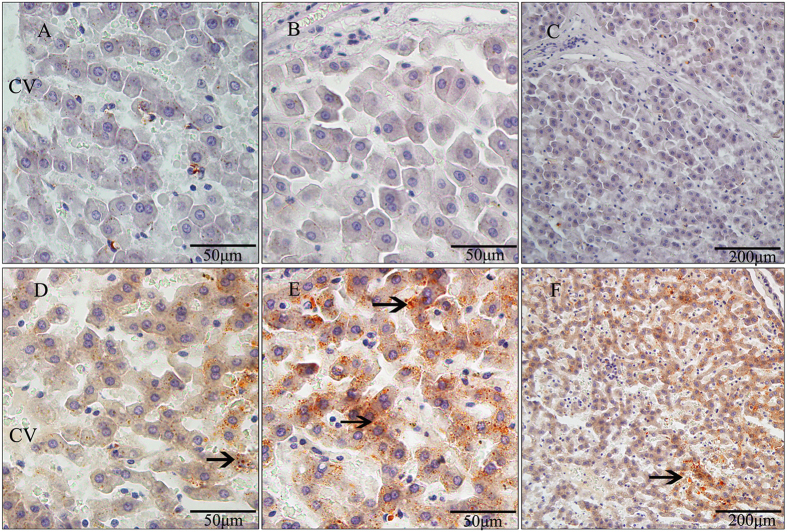
Fat lesions in the hepatic cells of each study group. High- and low-power magnification images show microvesicular steatosis in the hepatic cells of HFHSD minipigs (arrows). (**A**–**C**) Hepatic lobule (**A**), hepatic cells (**B**), and hepatic portal area (**C**) in the control group. (**D**–**F**): Hepatic lobule (**D**), hepatic cells (**E**), and hepatic portal area (**F**) in the HFHSD group. CV: central venous. (**A**,**B**,**D**,**E**): scale bar = 50 μm; (**C**,**F**): scale bar = 200 μm.

The overall steatohepatitis scores and the incidence of NAS (NAFLD activity score) were substantially higher in the HFHSD group than in the control group (Table 2), as determined according to the NASH Clinical Research Network Scoring System^24^ and our histopathological findings (Figs 4 and 5). Quantitative analysis of the distribution and type of lipid droplets with relation to the steatosis shows that all of the pigs in the HFHSD group had primarily microvesicular steatosis and that the steatosis was frequently observed in zone 1 and/or an azonal region. Although hepatocytic ballooning was not observed in either group, all of the pigs in the HFHSD group showed severe lobular and portal inflammation. Hepatic glycogen was observed in zones 1 and 3 of the minipigs in the HFHSD group (Figure S2). Using the NAS scoring system, four pigs in the HFHSD group had scores between 3 and 4. Thus, the condition of most of the pigs in this group ranged from a simple fatty liver to steatohepatitis.

### Characterization of microvesicular steatosis and inflammatory infiltration in the HFHSD group

In both groups, hepatic lobules were readily observable with the sinusoids radially distributed around the central vein. In the control group, the hepatocytes were neatly arranged and had large and obvious nuclei. No noticeable lipid droplets or intracellular lipid deposition were evident, while clear borders were present between the central vein and sinusoids (Fig. 4, minipig 157). In the HFHSD group, a diffuse distribution of lipids was observed in the hepatocytes and was associated with indistinct nuclei; however, no noticeable lipid droplets were evident. Mild hepatic iron overload was primarily present in the hepatocytes of zone 3 in the HFHSD group (Fig. 4, minipigs 126, 138, 140), while Sudan III staining revealed microvesicular steatosis in the hepatic cytoplasm (Fig. 5). The diffuse deposition and peroxidation of lipids within the hepatocytes were accompanied by an increase in inflammation and inflammatory cell infiltration (Fig. 4, minipigs 126, 138, 140), significant sinusoidal expansion and interspersion, and indistinct borders for the central vein (Fig. 4, zone 3). The hepatic sinusoid was abnormally dilated and showed severe lobular inflammatory cell infiltration by lymphocytes, Kupffer cells, eosinophils, and neutrophils (Fig. 4, zone 2). Moreover, there was a moderate presence of connective tissue and bile canaliculus hyperplasia in the portal area of the HFHSD minipigs with obvious portal inflammation. Periodic acid-Schiff staining revealed a large number of hepatic glycogen particles that were usually concentrated in the hepatocytes of zones 2 and 3 (Figure S2).

### Ultrastructural findings in the hepatocytes of the HFHSD group

The nuclei and organelles were the prominent structures in the hepatocytes of control livers (Figure S3, minipig 157). In the HFHSD group, fat vacuoles were abundant in the hepatocytes and displaced the nuclei near the cellular edge (Figure S3, minipigs 126, 138). The nuclei were irregular in shape and aggregated chromatin and/or an absent nucleolus were frequently observed. Major deformation of the mitochondria and other organelles was also evident. Many of the mitochondria were swollen and had a coarse appearance that was accompanied by blurred membranes, ruptured crista, and/or indistinct outlines. Additionally, in the HFHSD hepatocytes the rough endoplasmic reticulum was often fragmented and a decreased number of secondary lysosomes were observed.

### Transcriptome analysis of the livers from the HFHSD and control groups

**Figure 6:**
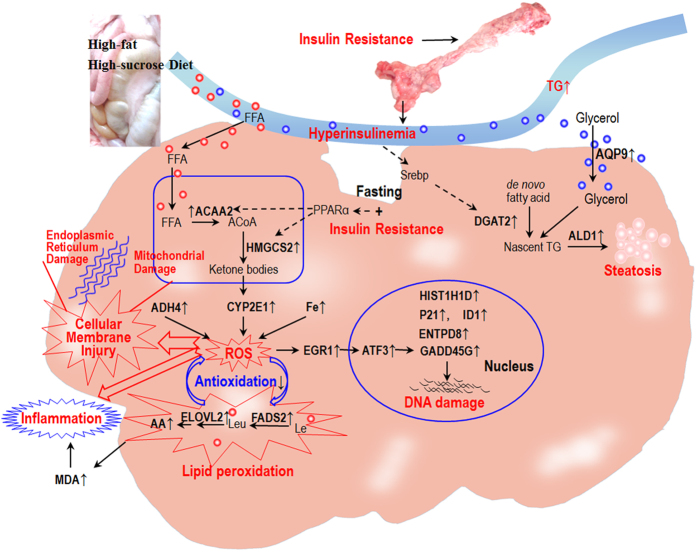
The characteristics of the pathogenesis of early stage non-alcoholic steatohepatitis. Insulin resistance leads to enlarged islets and hyperinsulinemia, which promotes the *de novo* synthesis of fatty acids and TG (DGAT2). These are deposited as small lipid droplets (ALD1) in hepatocytes. Insulin resistance shifts the energy supply from glucose to ketone bodies (HMGCS2) through peroxisome proliferator-activated receptor α in mitochondria. High concentrations of ketone bodies induce the overexpression of CYP2E1, which, when paired with iron overload and ADH4, mediates the production of ROS. Hyperinsulinemia promotes *de novo* synthesis of arachidonic acid (FADS2, ELOVL2). ROS production results in the peroxidation of arachidonic and linoleic acid and leads to malondialdehyde production, cellular membrane damage and inflammatory cell infiltration. ROS from lipid peroxidation induce the nuclear translocation of EGR1 and the activation of ATF3 and mediate DNA damage. Non-standard abbreviations: ACoA: acetyl coenzyme A; Le: linoleic acid; Leu: linoleic acid. Figure 6 was drawn by author Dr. Shulin Yang, and the photos in this figure come from experiments.

In total, we acquired 302.6 million reads from nine cDNA pig libraries, comprising 18.2 gigabases (Gb) of cDNA sequence. Approximately 82% of the sequenced reads (303 million mapped reads) were successfully aligned to the swine genome reference sequence (SGSC SusScr2). RNA-seq analysis detected 20,460 genes and 160 genes were differentially expressed between the livers of the HFHSD and control groups. Subsequent qRT-PCR analysis of 12 selected genes confirmed that their expression patterns were consistent with the RNA-seq results (Figure S4). Fifty-five differentially expressed genes were assigned to biological or signaling pathways (Fig. 6) including liver lipid deposition, energy metabolism, oxidation and antioxidation, DNA damage, inflammation, and fibrosis (Tables S1 and S2). The sequencing data sets are available in the NCBI Sequence Read Archive (access number, SRX197296).

### Energy supply and lipid metabolism related genes

A number of up-regulated genes were involved in fatty acid metabolism, including aquaporin 9 (a glycerol uptake channel in the liver). This gene product is involved in the uptake of glycerol^25^,^26^ in hepatocytes. Additionally, acetyl-CoA acyltransferase 2 and 3-hydroxy-3-methylglutaryl-CoA synthase 2 were up-regulated and are key enzymes that catalyze FFA decomposition into acetyl-coenzyme A^27^ and ketone body synthesis in the mitochondria^28^, respectively. The up-regulated fatty acid desaturase 2 mediates the desaturation of linoleic acid at the Δ5 position^29^, while fatty acid elongase 2 elongates long-chain fatty acids responsible for the *de novo* synthesis of arachidonic acid^30^. Diacylglycerol acyltransferase-2 and methyltransferase like 7B are involved in TG synthesis and deposition^31^,^32^. The down-regulated gene cortistatin is responsible for transporting medium- and long-chain acetyl-coenzyme A esters out of the peroxisome and mitochondria into the cytosol^33^. Three genes related to the synthesis and transport of creatine were also up-regulated: L-arginine:glycine amidinotransferase (*GATM*; creatine synthesis)^34^, solute-carrier family 6 member 8 (*SLC6A8*) (transport of creatine into and out of the cell)^35^ and mitochondrial creatine kinase 2 (transfer of high energy phosphates from the mitochondria to a cytosolic carrier via creatine)^36^. Creatine is both a reservoir for ATP-derived energy and a vehicle for moving energy from the mitochondria to the cytosol for consumption. The up-regulated expression of *GATM* and *SLC6A8* indicated that more creatine was being synthesized in the liver and transported to other tissues. This export can decrease the intracellular AMP:ATP ratio and repress the activation of adenosine monophosphate-activated protein kinase (AMPK) in these tissues during HFHSD obesity-induced insulin resistance^37^.

### Aggravated oxidative stress and rNeduced antioxidation related genes

Oxidative stress is an important pathogenic event in NASH and is indicated by the differential gene expression profiles reported in this study. There was significant up-regulation of the genes that encode the cytochrome P450 family 2 subfamily E polypeptide 1 (*CYP2E1*) and alcohol dehydrogenase 4 (*ADH4*) enzymes, which play important roles in the production of reactive oxygen species (ROS) in alcoholic fatty liver disease and NAFLD patients^38^,^39^. In addition, there was also differential expression of a number of genes involved in antioxidation, including the up-regulated genes paraoxonase-1 and glutathione S-transferase theta 1 as well as five down-regulated genes (e.g., thioldisulfide oxidoreductase).

### DNA damage related genes

In the HFHSD group, a dysfunction in DNA replication and transcription from oxidative stress was indicated by a number of up-regulated genes. These genes included the transcription factors for early growth response 1 (*EGR1*) and activating transcription factor 3 (*ATF3*), the cell cycle genes for growth arrest and DNA-damage-inducible gamma (*GADD45G*), ectonucleoside triphosphate diphosphohydrolase 8, and cyclin-dependent kinase inhibitor 1A (*P21*). They also included gene histone cluster 1 H1d (*HIST1H1D*), which is responsible for the suppression of DNA transcription. Genes related to apoptosis were also differentially expressed between the two groups, including two up-regulated genes: FBJ osteosarcoma oncogene, which promotes apoptosis, and docking protein 2, which inhibits cell differentiation. This group also included five down-regulated genes: desumoylating isopeptidase 2, cation transport regulator-like protein 1, annexin A5, and protease serine 23.

### Inflammatory and fibrosis related genes

A number of up-regulated genes involved in the inflammatory process were observed to be expressed in the NASH group, including those involved in inflammatory signaling pathways (e.g., major histocompatibility complex class II DM alpha, phospholipase C gamma 2, early growth response 2, paralemmin 3), the infiltration of inflammatory cells (myelin protein zero-like 2 and C-type lectin domain family 4 member F), and anti-inflammatory processes (secretoglobin family 1A member 1, interferon induced transmembrane protein 1). Mild hepatic perisinusoidal fibrosis and periportal fibrosis were indicated by a number of up-regulated genes in the HFHSD group; however, these genes showed smaller fold changes in expression (Table S1).

## Discussion

NAFLD is the hepatic manifestation of metabolic syndrome, which involves a cluster of related clinical features including insulin resistance, central obesity, increased TG and LDL-C, decreased HDL-C and hypertension^40^. In this study, Bama minipigs exhibited the typical characteristics of metabolic syndrome during a 23-month period on a HFHSD, including an approximately 3-fold change in body weight, the development of central and whole-body obesity, and an increase in visceral and subcutaneous fat. According to previous studies, the progression of the pathology can be divided into three stages^19^,^41^,^42^. The first stage encompasses the first four months, during which HDL-C, LDL-C, and TC increase rapidly, indicating that lipid metabolism has drastically changed. The second stage spans months 4 to 12 and does not involve any significant changes in any of the indices. The third stage ranges from month 12 to study completion. Serum insulin, the insulin resistance index (Figure S1 in) and TG increased gradually, and these increases were accompanied by decreasing HDL-C and glucose tolerance. Thus, we believe this stage is the beginning of insulin resistance. To date, no studies have reported experiments with Bama minipigs that received an overfeeding diet for more than 12 months. Studies of Gottingen^19^, Yucatan^20^, Ossabaw^21^,^22^, and Wuzhishan^23^ minipigs have also demonstrated rapid HDL-C and LDL-C increases in the initial stage and fluctuation within a narrow range in the following period. However, these studies may were too short to alter the TG, HDL-C, and IVGTT. The high-fat diet in our study promoted adipocyte hypertrophy and thus prevented TG uptake and increased cholesterol metabolism for cellular membrane formation in the first stage^41^. In the second stage of the experiment, the intake of fatty acids from the diet was balanced by the deposition of TG. The development of insulin resistance eventually inhibited the absorption of TG into the adipose tissues in the third stage, leading to increased serum TG concentrations and enlarged islets, resulting in hyperinsulinemia^42^. Therefore, to better understand the underlying mechanisms in the development of metabolic syndrome in minipig models, the second and third stages should be included in future studies, as well as analyses of the liver, pancreas, adipose and skeletal muscle tissues.

### Hyperinsulinemia promotes lipid deposition

Steatosis is characterized by the deposition of TG as lipid droplets into the cytoplasm of hepatocytes^8^. This study reports two interesting findings regarding the characteristics of liver steatosis in the HFHSD group. First, the amount of lipid deposition gradually decreased from the hepatic lobule edge to the central vein. This pattern of lipid deposition differed substantially from the pattern observed in rodent models, where large lipid droplets are deposited around the central vein^43^. Second, many of the lipid droplets were deposited as microvesicles but not as macrovesicles, which has also been observed in the Ossabaw minipig^22^. Furthermore, the microvesicular lipids were present as a number of small lipid droplets gathered on one side of the hepatocyte cytoplasm (Figure S3). Three different pieces of evidence imply that the deposited triacyglycerols were synthesized using *de novo* fatty acids in the liver and were not re-esterified from absorbed FFA in the HFHSD group: 1) hyperinsulinemia stimulates *de novo* fatty acid and nascent triacylglycerol synthesis through the up-regulation of the key enzyme diacylglycerol acyltransferase-2 through the activation of the transcription factor sterol regulatory element binding protein-1C^44^, 2) *DGAT1*, which is primarily involved in esterifying “old” fatty acid into TG observed between the two groups, was not differentially expressed, and 3) the serum FFA of the HFHSD group was significantly lower than that of the control group and was oxidized for energy supply but not re-esterified to TG. Furthermore, the gene for *ALD1* was up-regulated and *ALD* enhances lipid droplet formation and increases dispersion deposition^27^. We propose that the glucose was sequestered in the liver by hyperinsulinemia and was used to synthesize *de novo* fatty acids and used for nascent synthesis of TG, which were deposited as small lipid droplets in the hepatocytes to form the steatosis microvesicles (Fig. 6).

### Insulin resistance alters energy metabolism

When humans consume a high fat diet, the dietary FFAs transported to the hepatocytes have three major fates: oxidation for energy production or ketone body synthesis in the mitochondria, re-esterification to TG for storage in lipid droplets, or coupling to apolipoproteins for secretion as very-low density lipoproteins^3^. Fasting and insulin resistance promoted the up-regulation of acetyl-CoA acyltransferase 2 and 3-hydroxy-3-methylglutaryl-CoA synthase 2. This indicated that they are potentially mediated by peroxisome proliferator-activated receptor α^28^, implying that acetyl coenzyme A and ketone bodies were being produced in the livers of the HFHSD group (Fig. 6). The ketone bodies in the simple steatosis patient were significantly higher than in the normal group^45^. However, gene expression analysis of insulin production, glucose metabolism, and glycogenolysis showed that they were not correspondingly increased. Hence, the ketone bodies synthesized in the liver may have been exported to brain, muscle, and heart tissues as the primary energy supply rather than being consumed in glycogenolysis.

### Unmitigated oxidative stress mediates cellular injury and inflammation

Oxidative stress is essential for the progression of a fatty liver to NASH. By integrating the various types of collected data and data from previous studies, we were able to derive a comprehensive, interconnected model of the oxidative stress process from the *de novo* synthesis of polyunsaturated fatty acids to lipid peroxidation, ROS production, mitochondrial dysfunction, and inflammatory cell infiltration in minipigs (Fig. 6). In this model, insulin resistance-associated hyperinsulinemia promotes the *de novo* synthesis of arachidonic acid through the up-regulated expression of fatty acid elongase 2 and fatty acid desaturase 2 in the hepatocytes of the HFHSD group. This leads to an increased arachidonic acid:linoleic acid ratio and mediates oxidative stress and inflammation through polyunsaturated fatty acid peroxidation^46^. Moreover, the expression and activity of *CYP2E1* is increased as a result of the high concentration of ketone bodies during insulin resistance in NAFLD^10^, which is one of the most important causes of the disorder in the pig liver. *CYP2E1* can mediate the biotransformation of polyunsaturated fatty acids such as linoleic acid and arachidonic acid to generate ω-hydroxylated fatty acids, which can be further transformed by alcohol and aldehyde dehydrogenases to dicarboxylic fatty acids, which have deleterious intracellular effects^47^. *ADH4* has been suggested as a potential biomarker for NASH because of the alcohol produced by intestinal bacteria^9^, which metabolize a wide variety of substrates (e.g., ethanol) to produce ROS. Moreover, a study showed that elevated body iron stores have a detrimental effect on obesity-related conditions and that iron removal improves insulin sensitivity^48^. These findings show that iron plays a critical role in the formation of potent oxidants. Thus, in the HFHSD group, iron overload and the overexpression of the *CYP2E1* and *ADH4* genes may promote ROS production, which can drive the peroxidation of arachidonic acid and linoleic and lead to malondialdehyde production. Moreover, the reduction of glutathione peroxidase and total antioxidant capacity indicates a decrease in antioxidation capacity in the liver. Other potential factors such as endoplasmic reticulum stress, gut-derived signals and adipose tissue–derived factors may also impact the early stage of NASH in pigs. The oxidants produced by lipid peroxidation and reactive lipid aldehydes can cause liver toxicity through protein oxidation, enzyme inactivation, and damage to the cell membranes and nucleic acids (Fig. 6). Moreover, the NADPH oxidase-derived ROS from arachidonic acid peroxidation can induce the nuclear translocation of *EGR1*^49^, which in turn can stimulate the expression of the downstream genes *ATF3* and *GADD45G*^50^. ATF3 is a transcription factor involved in cell proliferation, apoptosis, and invasion^51^, while *GADD45G* is a member of the DNA damage-inducible gene family. In response to stress shock, *GADD45G* inhibits cell growth and induces apoptosis^50^. Therefore, the activated EGR1-ATF3-GADD45G pathway likely plays an important role in the arachidonic acid-mediated cytotoxicity in the HFHSD group.

The major mechanisms are summarized in Fig. 6 and were based on our current data and on the research of many previous studies. To demonstrate causative relationships between gene expression and the development of NASH in minipigs induced by HFHSD may require further study using knock-in or knock-out animals to up- or down-regulate the expression of related genes.

## Conclusions

In conclusion, this study showed that a long term high-fat, high-sucrose diet in Bama minipigs is a favorable animal model for understanding the mechanism of the gradual development of metabolic syndrome and NASH. The multiple integrated “hits” in the early stage presented as hyperinsulinemia and promoted the generation and deposition of nascent TG. They also increased the *de novo* synthesis of arachidonic acid. The insulin resistance shifted the energy supply to a high concentration of ketone bodies, induced *CYP2E1* overexpression, mediated ROS production with iron overload, induced *ADH4* overexpression and arachidonic acid peroxidation, induced cellular membrane damage, activated the DNA injury pathway EGR1-ATF3-GADD45G and triggered inflammation.

## Methods

### Minipigs and experimental design

The Bama minipig colony at Guangxi University was bred from a progenitor group of 2 males and 14 females and has been a closed colony since 1987. Twenty pregnant sows of the seventeenth generation were transferred to the Germplasm Resource Center for the Chinese Experimental Minipig at the Institute of Animal Sciences, Chinese Academy of Agricultural Sciences. The project was supported by a fund from the National Key S&T Project of the Tenth Five-year Plan from 2006 to 2007. Twelve 6-month-old Bama minipigs of either sex from the eighteenth generation were used in this study and received humane care according to the criteria outlined in the Guide for the Care and Use of Laboratory Animals, Institute of Animal Sciences, Chinese Academy of Agricultural Sciences. The procedures were approved by the Animal Care and Use Committee of the Germplasm Resource Center of Chinese Experimental Minipig (Permit Number: ACGRCM2008–012). All animals were housed in individual pens in controlled conditions (temperature, 18 °C–22 °C; relative air humidity, 30%–70%) and were fed twice daily on a restricted schedule and dietary dose [3% of body weight monthly; facility certification No.: SYXK (Beijing) 2008–007]. They had free access to water for 23 months. Six pigs were fed a control diet (control group), while another six pigs were fed a high-fat, high-sucrose diet (HFHSD) (53% control diet, 10% pork lard, and 37% sucrose; HFHSD group). At the end of the experiment, the animals were fasted overnight and euthanized with an overdose of ketamine and xylazine. The tissues were immediately weighed and frozen in liquid nitrogen and stored at −80 °C for subsequent analysis. The formalin-fixed and paraffin-embedded livers were processed with Sudan III and hematoxylin and eosin (H&E) staining.

### Phenotypes and serology assay

The body weights were recorded each month for both groups. Blood was obtained from the anterior vena cava of the animals after 16–20 hours of overnight fasting and centrifuged at 3500 rpm for 10 min at 4 °C. The serum glucose concentration was assayed using the immobilized glucose oxidase method. The serum insulin concentration was detected using a two-site immunometric assay with monoclonal antibodies as the immobilizing and detecting antibodies. The insulin resistance index was calculated with the formula HOMA-IR = (FINS × FPG)/22.5, where FINS is the fasting serum insulin and FPG is the fasting serum glucose. The serum triglyceride (TG), total cholesterol (TC), high-density lipoprotein cholesterol (HDL-C), β-hydroxybutyrate, acetoacetate and low-density lipoprotein cholesterol (LDL-C) measurements were performed using an autoanalyzer (7600, Hitachi, Tokyo, Japan). An intravenous glucose tolerance test (IVGTT) was performed in conscious pigs every two months after 16–20 hours of overnight fasting. Pigs were dosed intravenously via catheter with 1.2 mL glucose/kg body weight within 180 s in the ear. An acupuncture needle was used to draw blood from the edge of the other ear for glucose testing using a glucometer and strips (LifeScan, Inc., Milpitas, CA, USA) prior to injection and at 5, 10, 20, 30, 45, 60, 90, 120 minutes after injection. Alanine transaminase and aspartate aminotransferase, which are serum indicators of liver function, were assayed by the velocity method and by the end-point method. Superoxide dismutase levels were determined by a radioimmunoassay using radioimmunology kits (Santa Cruz Biotechnology, Santa Cruz, CA, USA). Serum iron concentrations were analyzed by colorimetric assays.

### Liver and pancreas pathology

The livers and pancreas tissues were fixed in 4% paraformaldehyde, dehydrated in a graded ethanol series, and embedded in paraffin. Paraffin sections with a thickness of 5 μm were stained with H&E for liver and islet pathology. Based on the H&E staining, the number of inflammatory cells in the liver per unit area was counted. A measurement of the pancreatic islet size was performed using Image-Pro Plus 6.0 (Media Cybernetics, Silver Springs, MD, USA). In addition, the tissue pieces of fixed livers were soaked in 15% and 30% sucrose solution and embedded in OTC. A freezing microtome (Leica CM 1900, Bensheim, Germany) was used to generate sections 8 μm thick. The sections were rinsed in distilled water and immersed in Sudan III solution for 5 to 10 min and counterstained with hematoxylin.

### Histopathological grading

The steatohepatitis score was calculated according to the NASH Clinical Research Network Scoring System. The steatosis was scored quantitatively as 0 (0–4%), 1 (5%–33%), 2 (34%–66%), or 3 (67%–100%). During the scoring process, the distribution and type of lipid droplets were also noted. Inflammation was classified as lobular or portal, and the lobular inflammation was scored as 0 (no foci), 1 (<2 foci per 200 × field), 2 (2–4 foci per 200 × field) or 3 (>4 foci per 200 × field). In addition, hepatic glycogen storage and portal/perisinusoidal fibrosis were separately graded as none, mild, or moderate depending on the level of glycogen. The pathology was scored in a double-blind manner by 2 independent pathologists.

### Liver ultrastructure

The liver ultrastructure was assessed by transmission electron microscopy. The liver samples that were stored in formaldehyde saline (4%) were cut into 1-mm × 3-mm pieces and fixed in glutaraldehyde (2.5%). The samples were subsequently fixed in osmium tetroxide (2%) and embedded in Spurr’s resin. Ultrathin sections were stained with uranyl acetate and lead citrate and viewed under an H-7500 (Japan) transmission electron microscope.

### Liver assays of fatty acids, ferric iron, and malondialdehyde

The pig livers were extracted with acid hydrolysis. The composition of the fat was determined using gas chromatography (GC-14C; Shimadzu, Tokyo, Japan). The relative retention times for each type of fatty acid were calculated according to fatty acid methyl ester standard solutions. A standard solution of 37 types of fatty acid methyl esters and a standard solution with a single fatty acid methyl ester were injected into the gas chromatograph, resulting in a number of response factors. The fatty acid methyl contents were determined according to the peak area of the chromatographic peak and the response factors. The relative compositions were calculated from three independent biological replicates. Liver malondialdehyde was analyzed using the thiobarbituric acid method, and Fe was analyzed by spectrophotometry.

### RNA-seq

The RNA library construction and sequencing was performed at Shanghai Biotechnology Corporation. The cDNA libraries were constructed following the TruSeq™ RNA Sample Preparation Guide (Illumina, San Diego, CA). Briefly, total RNA was isolated using an RNeasy Mini Kit (Qiagen), and mRNA was isolated with the Oligotex mRNA Mini Kit (Qiagen). The mRNAs were fragmented by incubation in Elute, Prime, Fragment Mix at 9 °C for 8 min to obtain 120–200 bp inserts. First-strand cDNA was synthesized with SuperScript II Reverse Transcriptase (Invitrogen) using random primers, and Ampure XP beads were used to isolate the double-stranded cDNA that was synthesized in Second Strand Master Mix. The adapter was ligated to the A-Tailing fragment and 12 cycles of PCR was performed to enrich the DNA fragments with adapter molecules at both ends and to increase the quantity of DNA in the library. Purified libraries were quantified using a Qubit^®^ 2.0 Fluorometer and validated using an Agilent 2100 Bioanalyzer to confirm the insert size and to calculate the molar concentration. A cluster was generated by cBot with the library and diluted to 10 pM and sequenced on the Illumina Genome Analyzer IIx for 75 cycles for 6 pigs (120, 126, 138, 140, 157, 161) and for 100 cycles for 3 pigs (144, 146, 159).

### Sequence analysis

Raw sequence data were preprocessed and assembled. This process included the original image data that was generated by the sequencing machine and converted to sequence data by base calling (Illumina pipeline CASAVA v1.8.0). The data were then filtered by standard QC criteria to remove all of the reads with the following parameters: reads that aligned to adaptors or primers with no more than two mismatches; reads with more than 10% unknown bases (N bases); and reads with more than 50% of low-quality bases (quality value ≤5) in a single read. The corresponding sequenced reads were mapped against the pig susScr2 genome by Tophat version 1.40. Swine (Sscrofa9.65) gene and transcript annotation files were obtained from the Ensembl database (http://asia.ensembl.org/info/data/ftp/index.html)^52^. The read count tables were generated from binary sequence alignment map (BAM) files using HTseq software (http://pypi.python.org/pypi/HTSeq). The fragments per kilobase of exon model per million mapped reads (FPKM) was calculated to estimate gene expression^53^. The read counts were adjusted by the edgeR software package using a one-scaling normalized factor for each sequenced library^54^. The DEseq software package was used to calculate the differences in gene expression levels between the HFHSD group (120, 126, 138, 140, 144, 146) and the control group (157, 159, 161). The fold changes of each gene between the HFHSD group (FPKM) and control group (FPKM) were log2-transformed. A gene was considered statically significant if the Benjamini and Hochberg-corrected P-value was less than 0.05 and the fold-change was greater than 1.5.

### Bioinformatics analysis of sequence data

KEGG (Kyoto Encyclopedia of Genes and Genomes) and GO (Gene Ontology analysis) mapping were performed based on annotation results. For GO analysis, contigs were categorized and statistically analyzed in terms of molecular function, cellular components, and biological processes. The results of KEGG and GO are shown in Table S2.

### Validation of RNA-Seq data by qRT-PCR

qRT-PCR was performed to validate the RNA-Seq results for the 12 gene transcripts. The PCR primers used in this study are listed in Table S3. The relative gene expression levels were calculated from the cycle number (Ct value). The threshold cycle (Ct) values were averaged from the values obtained from each reaction, and each gene was normalized to glyceraldehyde-3-phosphate dehydrogenase (GAPDH). These Ct values were averaged and the difference between the GAPDH Ct (Avg) and the gene of interest Ct (Avg) was calculated by 2^−ΔΔCT^, with ∆∆CT = (Ct treated—Ct control). The relative expression of the gene of interest was analyzed using the 2^−ΔΔCT^ method, and the data analysis used the results of three different experiments.

### Statistical analysis

The differences in body weight, serum biochemistry parameters and liver profiles between the two study groups were analyzed with the Statistical Package for the Social Sciences (SPSS) Version 18.0 for Windows (SPSS, Chicago, IL, USA). A one-way analysis of variance and the least significant difference (LSD) t-test were used for multiple comparisons. A P value of <0.05 was considered statistically significant. Data are presented as the mean ± the standard deviation.

## Additional Information

**How to cite this article**: Yang, S.-l. *et al.* Hyperinsulinemia shifted energy supply from glucose to ketone bodies in early nonalcoholic steatohepatitis from high-fat high-sucrose diet induced Bama minipigs. *Sci. Rep.* **5**, 13980; doi: 10.1038/srep13980 (2015).

## Supplementary Material

Supplementary Information

## Figures and Tables

**Table 1:** Body weight, serum biochemistry and liver profiles in sacrificed Bama minipigs.

	**HFHSD**	**Control**	**P value**
Body weight (kg)	140.28 ± 8.52	51.30 ± 5.85	<0.001^*^
Serum biochemistry			
Fasting glucose (mmol/L)	4.99 ± 1.11	6.62 ± 0.85	0.061
Fasting insulin (μIU/L)	28.32 ± 7.84	5.71 ± 0.39	0.001^*^
Total cholesterol (μmol/L)	3.41 ± 0.29	1.18 ± 0.38	0.002^*^
HDL-C (μmol/L)	0.97 ± 0.52	0.27 ± 0.07	0.038^*^
LDL-C (μmol/L)	1.32 ± 0.17	0.62 ± 0.17	0.004^*^
Triglyceride (μmol/L)	1.72 ± 0.32	0.44 ± 0.19	<0.001^*^
FFA (μmol/L)	215.63 ± 24.44	335.17 ± 95.50	0.027^*^
ALT (IU/L)	12.70 ± 1.35	18.00 ± 1.32	0.004^*^
AST (IU/L)	15.60 ± 1.98	31.00 ± 2.65	0.002^*^
SOD (IU/ml)	147.87 ± 19.59	188.82 ± 4.65	0.008^*^
Fe (μmol/L)	28.18 ± 6.96	18.35 ± 0.85	0.033^*^
Liver profile			
Ketone bodies (mmol/L)			
β-hydroxybutyrate	0.33 ± 0.03	0.02 ± 0.005	0.003^**^
Acetoacetate	0.019 ± 0.006	0.018 ± 0.001	0.166
Fatty acid			
Myristoleic (g/100 g)	0.023 ± 0.005	0.007 ± 0.012	0.118
Palmitic (g/100 g)	0.86 ± 0.08	0.58 ± 0.03	<0.001^*^
Palmitoleic (g/100 g)	0.055 ± 0.017	0.01 ± 0.01	0.020^*^
Stearic (g/100 g)	0.92 ± 0.05	0.86 ± 0.02	0.048^*^
Oleic (g/100 g)	0.92 ± 0.11	0.40 ± 0.02	<0.001^*^
Linoleic (g/100 g)	0.39 ± 0.08	0.68 ± 0.02	<0.001^*^
Arachidonic (g/100 g)	0.31 ± 0.16	0.12 ± 0.02	0.036^*^
MDA (nmol/ml)	2.24 ± 1.05	0.63 ± 0.27	0.019^*^
Fe (mg/100 g)	85.38 ± 28.83	45.68 ± 3.88	0.012^*^
GSH-PX (U/mg protein)	60.12 ± 24.42	119.51 ± 40.25	0.026^*^
T-AOC (U/mg protein)	0.21 ± 0.02	0.26 ± 0.04	0.048^*^
% of inflammatory cells	19.59 ± 2.60	6.37 ± 1.19	<0.05^*^
% of islets with an area of >10000 μm^2^	77.35 ± 1.76	22.63 ± 1.15	<0.01^*^

^*^Significant difference. Abbreviations: HDL-C: high-density lipoprotein cholesterol; LDL-C: low-density lipoprotein cholesterol; FFA: free fatty acids; ALT: glutamic-pyruvic transaminase; AST: glutamic-oxaloacetic transaminase; SOD: superoxide dismutase; Fe: Ferrum; MDA: malondialdehyde; GSH-PX: glutathione peroxidase; T-AOC: total antioxidant capacity.

**Table 2:** Comparison of histological scores and NASH between the study groups.

**Item**	**Category**	**Control group (n = 6)**	**HFHSD group (n = 6)**
**Steatosis**			
Grade	0 (0–4%)	4^*^	0
	1 (5%–33%)	2	1
	2 (34%–66%)	0	3
	3 (67%–100%)	0	2
Location	Zone 3	0	1
	Zone 1	0	3
	Azonal	0	2
Type	Macro-vacuolar	0	1
	Micro-vesicular	2	5
**Inflammation**			
Lobular inflammation	0 (no foci)	0	0
	1 (<2 foci)	All minipigs	0
	2 (2–4 foci)	0	4
	3 (>4 foci)	0	2
Portal inflammation		0	All minipigs
**Hepatic glycogen**			
Location	None	5	0
	Zone 3	0	4
	Zone 1	1	2
**NAS**			
	0–2	6	1
	3–4	0	4
	5–8	0	1

^*^Results are represented as the number of individuals from each group. *Abbreviations: NAS*: *nonalcoholic fatty liver disease activity score*.
