# Supplementary material for: Hyperinsulinemia shifted energy supply from glucose to ketone bodies in early nonalcoholic steatohepatitis from high-fat high-sucrose diet induced Bama minipigs
Source: Sci Rep. 2015 Sep 11;5:13980. doi: 10.1038/srep13980 (PMC4566077; doi:10.1038/srep13980)
Supplement: Supplementary Information [file srep13980-s1.pdf]

Hyperinsulinemia shifted energy supply from glucose to ketone bodies in early nonalcoholic steatohepatitis from high-fat high-sucrose diet induced Bama minipigs

Shu-lin Yang[1,†], Ji-han Xia[1,†], Yuan-yuan Zhang[2,†], Jian-gao Fan[3,†], Hua Wang[4], Jing Yuan[1,5], Zhan-zhao Zhao[2], Qin Pan[3], Yu-lian Mu[1], Lei-lei Xin[1], Yao-xing Chen[2,*], Kui Li[1,*]

[1]State Key Laboratory of Animal Nutrition, Institute of Animal Sciences, Chinese Academy of Agricultural Sciences, No.2 Yuanmingyuan West Road, Beijing, 100193, P.R. China

[2]College of Veterinary Medicine, China Agricultural University, No.2 Yuanmingyuan West Road, Beijing, 100193, P.R. China

[3]Department of Gastroenterology, Shanghai Key Laboratory of Children′s Digestion and Nutrition, Xinhua Hospital, Shanghai Jiaotong University School of Medicine, Shanghai, 200092, P.R. China

[4]Department of Oncology, The First Affiliated Hospital of Anhui Medical University, Hefei, 230032, Anhui, P.R. China

[5]College of Animal Science, Yangtz University, Jinzhou, 434023, Hubei, P.R. China

†Equal contributors

*Corresponding author

Contact Information

Address correspondence to Prof. Kui Li at Institute of Animal Sciences, Chinese Academy of Agricultural Sciences, No. 2 Yuanmingyuan West Road, Beijing 100193, P.R. China

Tel.: 86-010-62813822, Fax: 86-010-62813822, E-mail: likui@caas.cn

Supplementary Information

Figures

Figure S1: Insulin resistance index of the HFHSD and control groups during the 23-month feeding period. **Figure S2:** Periodic acid-Schiff stain showing glycogen accumulation in hepatic cells from minipigs fed the control or the HFHSD diet. **Figure S3:** Electron micrographs of the livers from the control group and the HFHSD group. **Figure S4:** Validation of the RNA-Seq data by qRT-PCR.

Tables

Table S1:The differential expressed genes involved in hepatocyte function. The differential expressed genes between the HFHSD and the control groups that are involved in liver lipid deposition, energy metabolism, oxidation and antioxidation, DNA damage, inflammation, and fibrosis. Gene ID, GenBank symbol, gene description, locus (chromosome location) and gene function of the pig genes in Ensembl database are listed. The average fragments per kilobase of exon model per

million mapped reads (FPKM) of the HFHSD and the control groups, and the fold changes between them are shown. The value of significant difference between the HFHSD and the control groups was calculated by Q-values and P-values; the significant ‘yes’ indicated Q-value < 0.05.

Table S2 - GO and KEGG analysis

KEGG (Kyoto Encyclopedia of Genes and Genomes) and GO (Gene Ontology analysis) mapping were performed based on annotation results. For GO analysis, contigs were categorized and statistically analyzed in terms of molecular function, cellular component, and biological process.

Table S3 - Primer pairs selected for analysis by qRT PCR

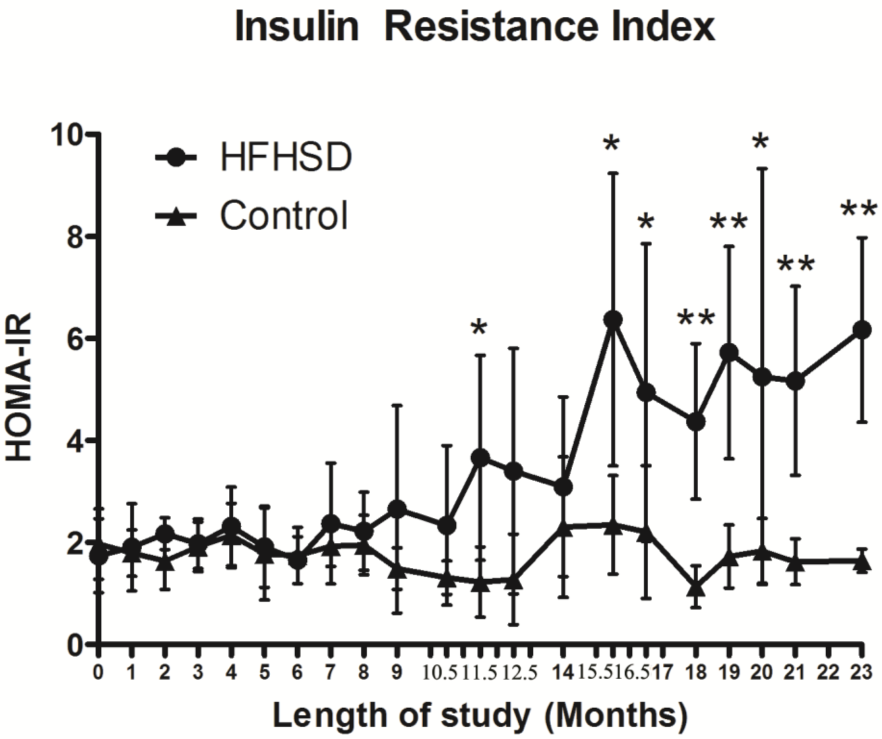

Figure S1. Insulin resistance index of the HFHSD and control groups during the 23 months of the study. *P < 0.05 and **P < 0.01 indicate the significance levels between the two groups. The Insulin Resistance Index of the HFHSD group was significantly higher than that of the control group during the last seven months of the study, which indicates that the HFHSD group had lower insulin sensitivity than did the control group.

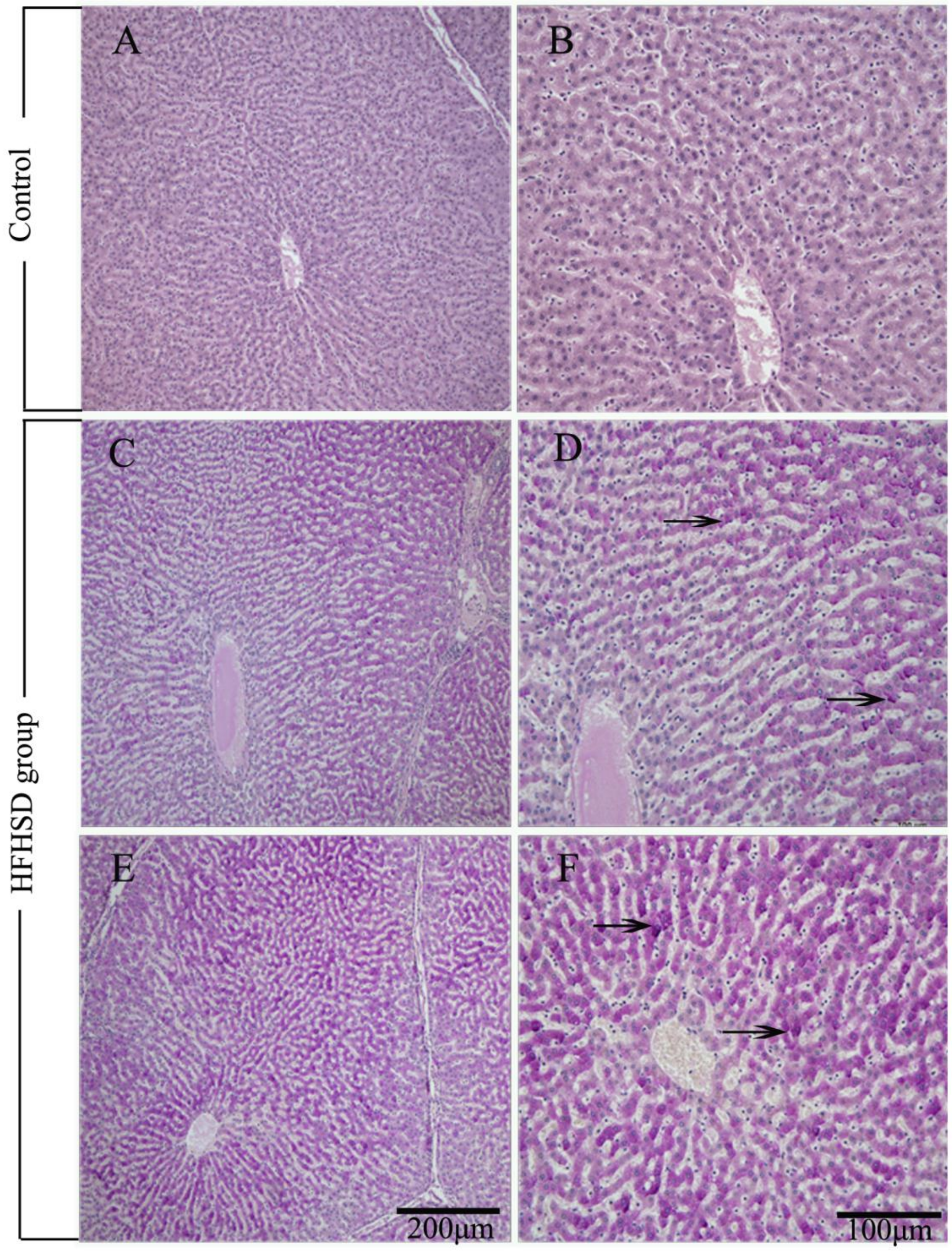

Figure S2. Periodic acid-Schiff stain showing glycogen accumulation in hepatic cells from minipigs fed control or HFHSD diets. A, C, E: Hepatic lobule; B, D, F: central vein. A, E, C: scale bar = 200 μm; B, D, F: scale bar =100 μm.

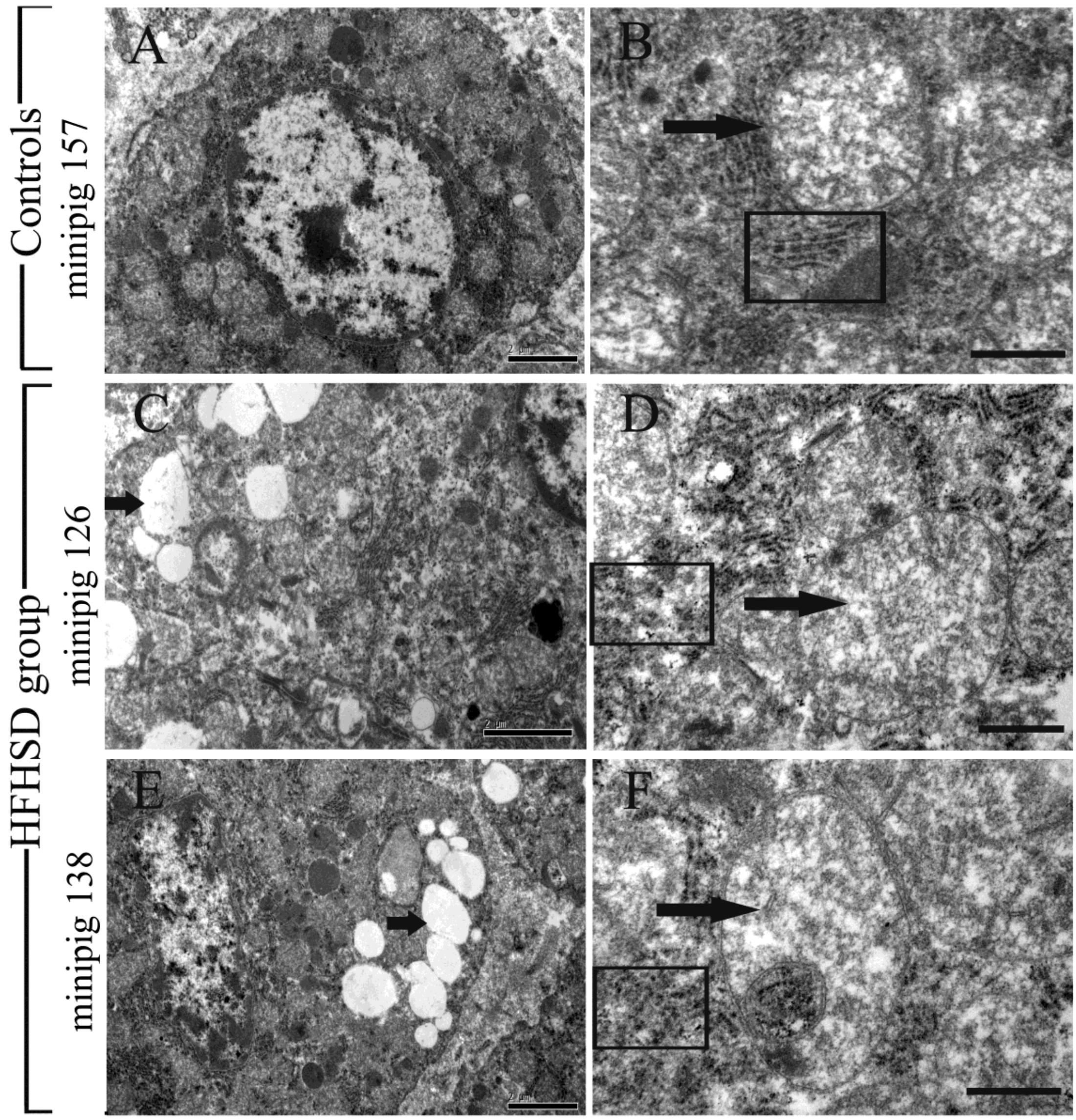

Figure S3. Electron micrographs of livers from the control group (minipig 157) and the HFHSD group (minipig 126 and minipig 138). HFHSD pigs had abnormal liver ultrastructure (electron microscopy). C and E show abundant fat vacuoles in the hepatocytes of HFHSD groups (arrow heads). D and F show mitochondria with irregular boundaries and became swollen and coarse accompanied by cracked crista or/ and unclear outline (arrow heads). In black boxes of D and F, there are endoplasmic reticulums fragmentated with increased free ribosomes. A, C, E scale = 2 μm; B, D, F scale = 0.5 μm.

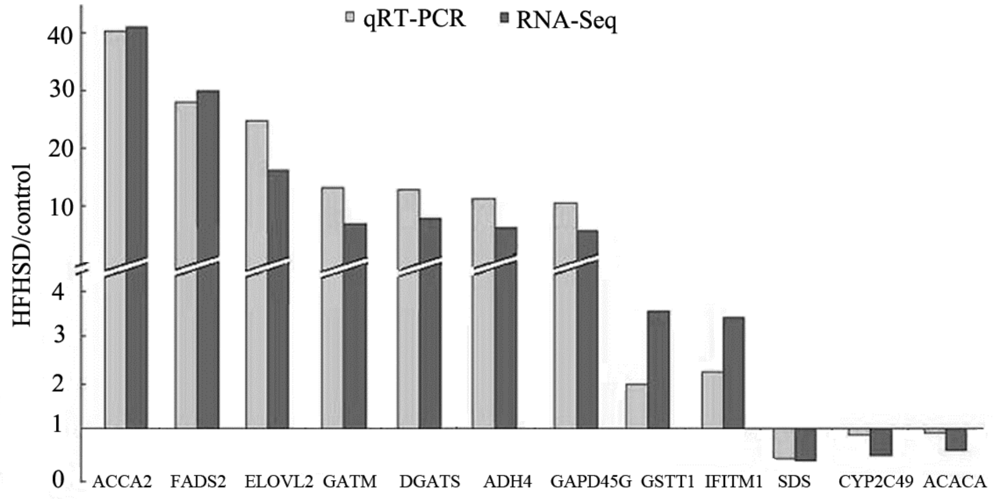

Figure S4. Validation of RNA-Seq data by qRT-PCR. Twelve genes up- or down-regulated in the livers of HFHSD minipigs and associated with lipid metabolism, chemokines, and immune response were selected for validation. Data are presented as the fold difference between the HFHSD and control groups. Each gene was normalized to glyceraldehyde-3-phosphate dehydrogenase (GAPDH).

Table S1: The differential expressed genes involved in hepatocyte function.

Gene_id	GenBank Symbol	Gene description	FPKM (HFHSD)	FPKM (control)	Fold change (HFHSD/control)	P-value	Q-value	Significant
**Energy supply and lipid metabolism**								
ENSSSCG00000004510	ACAA2	acetyl-CoA acyltransferase 2	409.85	9.48	5.43	2.33E-16	2.84E-13	yes
ENSSSCG00000004597	AQP9	aquaporin 9	68.06	13.18	2.37	1.31E-07	4.45E-05	yes
ENSSSCG00000014128	CKMT2	creatine kinase 2	4.48	0.82	2.45	0.0004468	0.0343026	yes
ENSSSCG00000015391	CROT	cortistatin	114.54	568.14	-2.31	1.98E-05	0.00274385	yes
ENSSSCG00000014863	DGAT2	diacylglycerol O-acyltransferase 2	145.51	23.90	2.61	1.79E-06	0.00039579	yes
ENSSSCG00000001045	ELOVL2	fatty acid elongase 2	49.74	3.73	3.74	2.97E-10	2.01E-07	yes
ENSSSCG00000013072	FADS2	fatty acid desaturase 2	224.63	8.08	4.80	1.40E-14	1.79E-11	yes
ENSSSCG00000004672	GATM	L-arginine:glycine amidinotransferase	563.97	106.39	2.41	1.40E-05	0.00212744	yes
ENSSSCG00000006716	HMGCS2	3-hydroxy-3-methylglutaryl-CoA synthase 2	78.20	11.01	2.83	2.84E-07	8.62E-05	yes
ENSSSCG00000000357	METTL7B/ ALDI	methyltransferase like 7B	332.60	74.10	2.17	5.30E-05	0.00632651	yes
ENSSSCG00000012771	SLC6A8	solute-carrier family 6 member 8 (MCT6)	40.34	10.99	1.88	2.37E-05	0.00316799	yes
ENSSSCG00000017215	SLC16A5	solute carrier family 16, member 5	33.73	3.86	3.13	7.04E-09	3.38E-06	yes
**Oxidative and antioxidation**								

ENSSSCG00000009184	ADH4	alcohol dehydrogenase 4	753.02	152.55	2.30	2.58E-06	0.000530865	yes
ENSSSCG00000004578	ANXA2	annexin-2	42.27	148.52	-1.81	8.56E-05	0.00929551	yes
ENSSSCG00000010780	CYP2E1	cytochrome P450, family 2, subfamily E, polypeptide 1	2522.05	90.65	4.80	2.22E-16	3.65E-13	yes
ENSSSCG00000003158	DHDH	dihydrodiol dehydrogenase	45.16	184.01	-2.03	3.28E-05	0.00420215	yes
ENSSSCG00000010803	GLRX2	glutaredoxin 2	28.82	112.84	-1.97	1.93E-05	0.0027371	yes
ENSSSCG00000010064	GSTT1	glutathione S-transferase theta 1	64.96	18.73	1.79	0.00034095	0.0278468	yes
ENSSSCG00000015332	PON1	paraoxonase 1	267.77	80.30	1.74	0.00012808	0.0129661	yes
ENSSSCG00000003914	PRDX1	peroxiredoxin 1	153.20	507.14	-1.73	9.62E-05	0.0102533	yes
ENSSSCG00000000415	SDR9C7	short chain dehydrogenase/reductase family 9C, member 7	7.16	30.30	-2.08	0.00063201	0.0452061	yes
**DNA damage**								
ENSSSCG00000009097	ANXA5	annexin A5	24.19	70.16	-1.54	0.00037552	0.0298244	yes
ENSSSCG00000015595	ATF3	activating transcription factor 3	57.13	8.84	2.69	2.43E-07	7.57E-05	yes
ENSSSCG00000001565	CDKN1A	cyclin-dependent kinase inhibitor 1A (p21, Cip1)	51.01	15.55	1.71	0.00033417	0.0274878	yes
ENSSSCG00000004754	CHAC1	cation transport regulator homolog 1	0.66	6.73	-3.34	8.82E-05	0.00949643	yes
ENSSSCG00000009612	DOK2	docking protein 2	19.85	3.82	2.38	2.11E-05	0.00289956	yes
ENSSSCG00000014336	EGR1	early growth response 1	102.00	26.17	1.96	1.08E-05	0.00175316	yes
ENSSSCG00000005852	ENTPD8	ectonucleoside triphosphate diphosphohydrolase 8	29.68	4.30	2.79	5.79E-07	0.000162544	yes

ENSSSCG00000002383	FOS	FBJ murine osteosarcoma viral oncogene homolog	258.20	50.68	2.35	1.82E-06	0.00039579	yes
ENSSSCG00000009585	GADD45G	growth arrest and DNA-damage-inducible, gamma	136.75	31.77	2.11	2.73E-06	0.00054167	yes
ENSSSCG00000001137	HIST1H1D	histone cluster 1, H1d	63.49	9.02	2.82	4.30E-08	1.65E-05	yes
ENSSSCG00000004452	PRSS23	protease, serine, 23	4.80	0.46	3.38	0.0001889	0.0176863	yes
**Inflammation**								
ENSSSCG00000008320	CLEC4F	C-type lectin domain family 4, member F	23.89	3.34	2.84	1.54E-07	5.08E-05	yes
ENSSSCG00000010224	EGR2	early growth response 2	6.94	1.10	2.66	5.76E-05	0.00676443	yes
ENSSSCG00000014564	IFITM1	interferon induced transmembrane protein 1	81.79	21.91	1.90	4.17E-05	0.0050524	yes
ENSSSCG00000015091	MPZL2	myelin protein zero-like 2	9.96	1.48	2.75	1.17E-05	0.00181586	yes
ENSSSCG00000013767	PALM3	paralemmin 3	106.90	24.62	2.12	1.90E-06	0.000404346	yes
ENSSSCG00000002688	PLCG2	phospholipase C, gamma 2 (phosphatidylinositol-specific)	5.37	1.20	2.16	5.33E-05	0.00632651	yes
ENSSSCG00000013060	SCGB1A1	secretoglobin, family 1A, member 1 (uteroglobin)	131.26	26.43	2.23	0.00032904	0.0272609	yes
**Fibrosis**								
ENSSSCG00000012138	ACE2	angiotensin I converting enzyme 2	27.24	5.46	2.32	5.15E-07	0.000151388	yes
ENSSSCG00000006106	CDH17	cadherin 17, LI cadherin (liver-intestine)	2.15	0.34	2.66	0.00045965	0.0350552	yes
ENSSSCG00000010728	CPXM2	carboxypeptidase X 2	87.20	16.16	2.43	3.82E-06	0.000698269	yes
ENSSSCG00000001085	DCDC2	doublecortin domain containing	3.36	0.39	3.09	0.0003734	0.0298244	yes

		2				7		
ENSSSCG00000010922	ELF3	E74-like factor 3	7.18	1.01	2.83	2.44E-05	0.00323169	yes
ENSSSCG00000009613	EPB49	erythrocyte membrane protein band 4.9 (dematin)	16.81	4.17	2.01	6.87E-05	0.00783255	yes
ENSSSCG00000007586	FSCN1	fascin homolog 1, actin-bundling protein	5.30	1.21	2.14	0.0004142 3	0.0322703	yes
ENSSSCG00000012376	GDPD2	glycerophosphodiester phosphodiesterase 2	8.00	1.18	2.77	1.58E-05	0.00233827	yes
ENSSSCG00000007227	ID1	inhibitor of DNA binding 1	79.03	15.38	2.36	8.88E-07	0.000237897	yes
ENSSSCG00000003709	LAMA3	laminin, alpha 3	2.86	0.83	1.79	0.0004147 3	0.0322703	yes
ENSSSCG00000012852	MUPCDH	cadherin-related family member 5	6.12	0.38	4.01	6.79E-08	2.37E-05	yes
ENSSSCG00000002744	PKD1L3	polycystic kidney disease 1 like 3	80.38	16.94	2.25	4.64E-08	1.72E-05	yes
ENSSSCG00000000181	RND1	Rho family GTPase 1	50.43	9.26	2.45	9.90E-07	0.000253289	yes
ENSSSCG00000007467	TMEM189	transmembrane protein 189	32.98	6.48	2.35	7.96E-06	0.0013689	yes

Table S2 - GO and KEGG analysis

#Term	Database	Id	Sample number	P-Value	Corrected P-Value
Drug metabolism - cytochrome P450	KEGG PATHWAY	ssc00982	7 / 70	3.98E-06	0.000242653
Metabolism of xenobiotics by cytochrome P450	KEGG PATHWAY	ssc00980	6 / 70	8.03E-05	0.002447937
Arginine and proline metabolism	KEGG PATHWAY	ssc00330	5 / 70	0.000251767	0.005119258
Arachidonic acid metabolism	KEGG PATHWAY	ssc00590	5 / 70	0.000707082	0.010067146
Metabolic pathways	KEGG PATHWAY	ssc01100	24 / 70	0.000825176	0.010067146
Renin-angiotensin system	KEGG PATHWAY	ssc04614	3 / 70	0.001103884	0.011222817
Pentose and glucuronate interconversions	KEGG PATHWAY	ssc00040	3 / 70	0.001344859	0.011719483
Fatty acid metabolism	KEGG PATHWAY	ssc00071	4 / 70	0.00210786	0.014286608
Valine, leucine and isoleucine degradation	KEGG PATHWAY	ssc00280	4 / 70	0.00210786	0.014286608
Histidine metabolism	KEGG PATHWAY	ssc00340	3 / 70	0.002628604	0.016034484
Biosynthesis of unsaturated fatty acids	KEGG PATHWAY	ssc01040	2 / 70	0.010063034	0.052210368
HTLV-I infection	KEGG PATHWAY	ssc05166	8 / 70	0.010270892	0.052210368
Circadian rhythm - mammal	KEGG PATHWAY	ssc04710	2 / 70	0.014012379	0.065736431
Lysine degradation	KEGG PATHWAY	ssc00310	3 / 70	0.01508705	0.065736431
Ascorbate and aldarate metabolism	KEGG PATHWAY	ssc00053	2 / 70	0.018521421	0.075320447
Glycerolipid metabolism	KEGG PATHWAY	ssc00561	3 / 70	0.019883318	0.075805151

Retinol metabolism	KEGG PATHWAY	ssc00830	3 / 70	0.021199097	0.076067348
Glycolysis / Gluconeogenesis	KEGG PATHWAY	ssc00010	3 / 70	0.025427613	0.086171356
beta-Alanine metabolism	KEGG PATHWAY	ssc00410	2 / 70	0.041499781	0.126574332
Linoleic acid metabolism	KEGG PATHWAY	ssc00591	2 / 70	0.041499781	0.126574332
Protein digestion and absorption	KEGG PATHWAY	ssc04974	3 / 70	0.046519749	0.134000535
Asthma	KEGG PATHWAY	ssc05310	2 / 70	0.048328062	0.134000535
Prion diseases	KEGG PATHWAY	ssc05020	2 / 70	0.051885306	0.13550184
Leishmaniasis	KEGG PATHWAY	ssc05140	3 / 70	0.054993999	0.13550184
Glycine, serine and threonine metabolism	KEGG PATHWAY	ssc00260	2 / 70	0.055533541	0.13550184
Propanoate metabolism	KEGG PATHWAY	ssc00640	2 / 70	0.059269549	0.139055481
Pyruvate metabolism	KEGG PATHWAY	ssc00620	2 / 70	0.070973085	0.150895761
Cell adhesion molecules (CAMs)	KEGG PATHWAY	ssc04514	4 / 70	0.071970908	0.150895761
Steroid hormone biosynthesis	KEGG PATHWAY	ssc00140	2 / 70	0.0750294	0.150895761
Graft-versus-host disease	KEGG PATHWAY	ssc05332	2 / 70	0.0750294	0.150895761
ErbB signaling pathway	KEGG PATHWAY	ssc04012	3 / 70	0.079125678	0.150895761
Tryptophan metabolism	KEGG PATHWAY	ssc00380	2 / 70	0.079158432	0.150895761
Mineral absorption	KEGG PATHWAY	ssc04978	2 / 70	0.083357369	0.154084833
Fat digestion and absorption	KEGG PATHWAY	ssc04975	2 / 70	0.08762346	0.157206796
Allograft rejection	KEGG PATHWAY	ssc05330	2 / 70	0.091954019	0.160262718
Type I diabetes mellitus	KEGG PATHWAY	ssc04940	2 / 70	0.109869269	0.186167372
Rheumatoid arthritis	KEGG PATHWAY	ssc05323	3 / 70	0.113094084	0.186452409
Intestinal immune network for IgA production	KEGG PATHWAY	ssc04672	2 / 70	0.123859709	0.193729288
Staphylococcus aureus infection	KEGG PATHWAY	ssc05150	2 / 70	0.123859709	0.193729288
Autoimmune thyroid disease	KEGG PATHWAY	ssc05320	2 / 70	0.138256002	0.205697954
Glioma	KEGG PATHWAY	ssc05214	2 / 70	0.138256002	0.205697954

Viral myocarditis	KEGG PATHWAY	ssc05416	2 / 70	0.178205515	0.258822296
Toxoplasmosis	KEGG PATHWAY	ssc05145	3 / 70	0.186511447	0.264586006
p53 signaling pathway	KEGG PATHWAY	ssc04115	2 / 70	0.193627591	0.268438251
Antigen processing and presentation	KEGG PATHWAY	ssc04612	2 / 70	0.204005976	0.276541435
Peroxisome	KEGG PATHWAY	ssc04146	2 / 70	0.209219629	0.277443421
Hepatitis C	KEGG PATHWAY	ssc05160	3 / 70	0.219531244	0.284923529
ECM-receptor interaction	KEGG PATHWAY	ssc04512	2 / 70	0.246038902	0.312674438
Phagosome	KEGG PATHWAY	ssc04145	3 / 70	0.27297813	0.339829917
Amoebiasis	KEGG PATHWAY	ssc05146	2 / 70	0.304262954	0.371200804
Axon guidance	KEGG PATHWAY	ssc04360	2 / 70	0.351399213	0.42030102
Leukocyte transendothelial migration	KEGG PATHWAY	ssc04670	2 / 70	0.382256731	0.437554535
Cell cycle	KEGG PATHWAY	ssc04110	2 / 70	0.382256731	0.437554535
Chagas disease (American trypanosomiasis)	KEGG PATHWAY	ssc05142	2 / 70	0.387343359	0.437554535
Systemic lupus erythematosus	KEGG PATHWAY	ssc05322	2 / 70	0.402495566	0.446404173
Tight junction	KEGG PATHWAY	ssc04530	2 / 70	0.412502275	0.449332835
Pathways in cancer	KEGG PATHWAY	ssc05200	4 / 70	0.487306733	0.521503697
Purine metabolism	KEGG PATHWAY	ssc00230	2 / 70	0.555690457	0.584433066
Tuberculosis	KEGG PATHWAY	ssc05152	2 / 70	0.600383432	0.618281141
Influenza A	KEGG PATHWAY	ssc05164	2 / 70	0.608145385	0.618281141
MAPK signaling pathway	KEGG PATHWAY	ssc04010	2 / 70	0.788746954	0.788746954

Table S3 - Primer pairs selected for analysis by qRT PCR

Gene	Forward primer	Reverse primer
ACAA2	ACGGTTAATAGGCTCTGTGGC	GCTCATGCTTTCAGTTCCTCCAC
ELOVL2	TGTTTCCGTCCATGCACAAGTAT	CATGTACGAGGACTGGAAGATGA
FADS2	CTTAAAGGGTGCCTCTGCCAACT	GGTATTTCAGCTTCTTCTTGCCGTAC
ADH4	GGAAGCAAACAAACCGCTCAACA	ATGGCCGAGGATCACTGGGAAAA
IFITM1	GTGCTGCCTGGGCTTCGTGGCTTTC	CAGTGGCTCCGATGGTCAGAATG
ACACA	GAGGAATACCCGTGGGAGTAGTTG	CCTGCTGGATTATCTTGGCTTCA
SDS	GCATCCCTGCCTTGATTGTCGTG	GACCCAGTTTGAGTTGTTCTTCACCAG
GATM	ATGGGCATTGAATGGATGCGTAA	GGGTCGGTCAGGGTTGGAAAGTA
DGAT2	AGGCACCGGCTCCAGCAT	CACTCCCAGCACGAGGAAAGAC
GAPD45G	TACGAGTCCGCCAAAGTCCTGAATG	CAGAACGCCTGGATCAAAGTGAAGTG
GSTT1	TGCTGCCCTCTTCCCACCCT	GCAGTAAAGGCAAAGTTAGCAAACAGAC

CYP2C49	CCTCGGGACTTCATTGATTGTTT	GAGCCCATATCTCAGGGTGGTAC

Abbreviation: ACAA2: acetyl-CoA acyltransferase 2; ELOVL2: fatty acid elongase 2; FADS2: fatty acid desaturase 2; ADH4: alcohol dehydrogenase 4; IFITM1: interferon-induced transmembrane protein 1; ACACA: acetyl-CoA acyltransferase 2; SDS: L-serine deaminase; GATM: L-arginine: glycine amidinotransferase; DGAT2: diacylglycerol O-acyltransferase 2; GAPD45G: growth arrest and DNA-damage-inducible, gamma; GSTT1: glutathione S-transferase theta 1; CYP2C49: Cytochrome P450 2C49
